# Biosynthesis and Transport of Nucleotide Sugars for Plant Hemicellulose

**DOI:** 10.3389/fpls.2021.723128

**Published:** 2021-11-12

**Authors:** Wenjuan Zhang, Wenqi Qin, Huiling Li, Ai-min Wu

**Affiliations:** ^1^State Key Laboratory for Conservation and Utilization of Subtropical Agro-Bioresources, Guangdong Key Laboratory for Innovative Development and Utilization of Forest Plant Germplasm, College of Forestry and Landscape Architecture, South China Agricultural University, Guangzhou, China; ^2^Guangdong Key Laboratory for Innovative Development and Utilization of Forest Plant Germplasm, College of Forestry and Landscape Architectures, South China Agricultural University, Guangzhou, China; ^3^Guangdong Laboratory of Lingnan Modern Agriculture, Guangzhou, China

**Keywords:** hemicellulose, nucleotide sugar, transporter, biosynthesis, cell wall

## Abstract

Hemicellulose is entangled with cellulose through hydrogen bonds and meanwhile acts as a bridge for the deposition of lignin monomer in the secondary wall. Therefore, hemicellulose plays a vital role in the utilization of cell wall biomass. Many advances in hemicellulose research have recently been made, and a large number of genes and their functions have been identified and verified. However, due to the diversity and complexity of hemicellulose, the biosynthesis and regulatory mechanisms are yet unknown. In this review, we summarized the types of plant hemicellulose, hemicellulose-specific nucleotide sugar substrates, key transporters, and biosynthesis pathways. This review will contribute to a better understanding of substrate-level regulation of hemicellulose synthesis.

## Introduction

The plant cell wall, primarily composed of cellulose, hemicellulose, pectin, and lignin, serves a variety of functions, including protection, support, material transport, and information exchange (Pauly and Keegstra, 2008). The plant cell wall is composed of into three layers: the middle lamella, the primary wall, and the secondary wall (Pauly and Keegstra, 2008). Middle lamella makes first thin layer mainly rich with pectin and is formed to connect two adjacent cells. The plant subsequently produces nucleosides and their metabolites *via* photosynthesis and adds them to the middle lamella, forming a flexible and elastic the primary cell wall. After cell growth ceases, the cell wall thickens in the inside and accumulate cellulose, hemicellulose, and lignin to form secondary cell wall.

**Figure 1:**
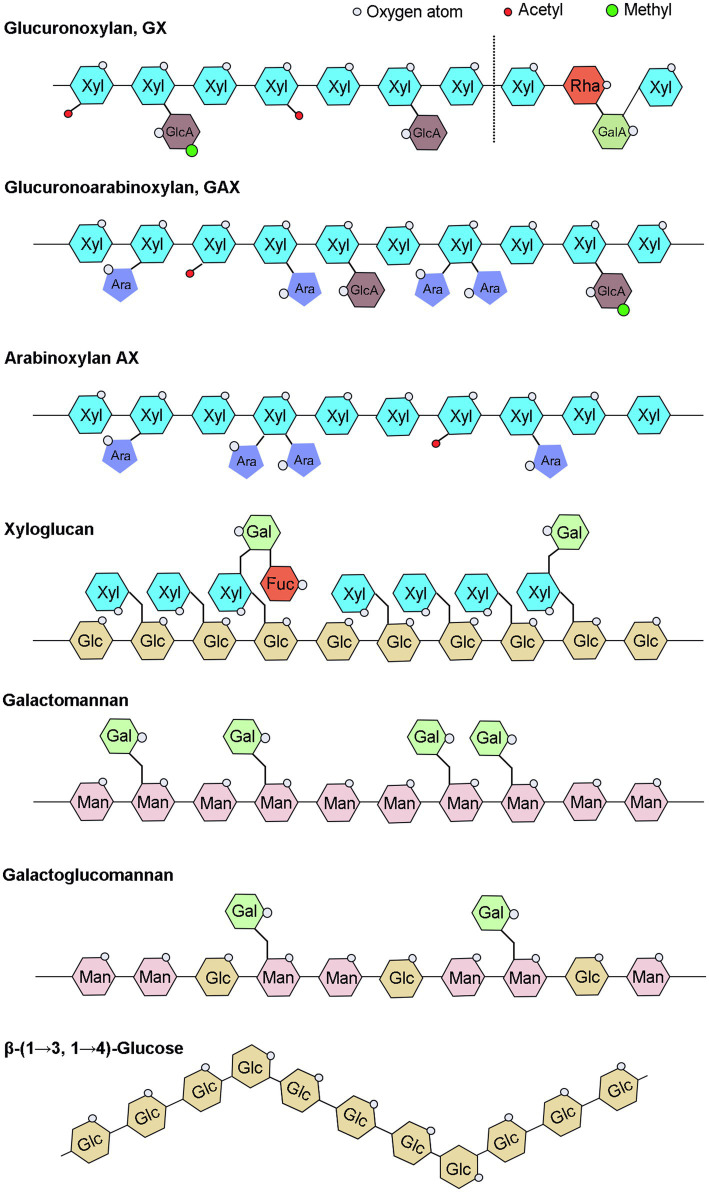
Schematic illustration of hemicelluloses. UDP-Glc, Uridine diphosphate glucose; UDP-GlcA, UDP-glucuronic acid; UDP-Xyl, UDP-xylose; UDP-Gal, UDP-galactose; UDP-GalA, UDP-galacturonic acid; UDP-Ara, UDP-arabinose; UDP-Rha, UDP-rhamnose; GDP-Man, guanosine diphosphate-mannose; and GDP-Fuc, GDP-fucose.

Hemicellulose, a broad term for a group of complex glycans, is a major component of plant cell walls and one of the most essential modern chemical raw materials for fuel. Hemicellulose widely used in variety of other fields, including as food additives in food industry and as plasticizer, drug delivery agent in medicinal industry (Qaseem et al., 2021). Hemicelluloses have various distinct structures, primarily xylan, xyloglucan, mannan, β-(1→3, 1 →4)-glucan, and their derivatives, and their content and detailed structure vary greatly depending on plant species and growth phases (Scheller and Ulvskov, 2010; Figure 1). Xylan is the most abundant type of hemicelluloses in broad-leaved woods, cereals, and dicotyledonous herbs. Mannan is mainly found in gymnosperms, and xyloglucan is a minor hemicellulose component of all terrestrial plants, including mosses (Scheller and Ulvskov, 2010). β-(1→3, 1→4)-glucans are less abundant in many plants than other hemicelluloses, but are abundant in grasses and have received less attention.

Hemicelluloses are a general term for heteropolysaccharides composed of two or more free monosaccharides linked in various ways, such as xylose, glucose, mannose, and galactose. Xylose is the main hemicellulose monosaccharide in grasses and hardwood, whereas arabinose, galactose, and mannose are the principal hemicellulose monosaccharides in softwood, plant seeds, endosperms, and fruits (Gibeaut et al., 2005; Schadel et al., 2010). Hemicellulose is composed of several different types of five-carbon sugars [β-D-xylose (Xyl), α-l-arabinose (Ara), α-l-rhamnose (Rha), and α-l-fucose (Fuc)], six-carbon sugars [β-D-glucose (Glu), β-D-mannose (Man), and α-D-galactose (Gal)], and glyoxalate [UDP-glucuronic acid (UDP-GlcA) and UDP-galacturonic acid (UDP-GalA)] monomers (Schadel et al., 2010).

Among them, UDP-Glc and GDP-Man work as upstream originators then are further catalyzed and converted to other nucleotide sugars by a series of 4-isomerase, 3,5-isomerase, 4-reductase, 4,6-dehydratase, 6-dehydrogenase, and decarboxylase (Reiter, 2008). Many other nucleotide sugars are abundant in plant cell walls, but there is no clear evidence that they are structural component of hemicelluloses. Studying and comprehending the process of the nucleotide sugars synthesis, interconversion, and transport can help in analysis of structures and functions, as well as the regulation of hemicellulose. In this review, we mainly encompass the synthesis and transportation of hemicellulose nucleotide sugars.

## Main Structures and Functions of Plant Hemicellulose

The xylan backbone consists of xylose residues linked by a β-(1→4) glycosidic bond, with reducing tetrasaccharide structure: β-D-xylose-(1→3)-α-l-rhamnose-(1→2)-α-D-galacturonic acid-(1→4)-D-xylose at the end of the xylan backbone in dicots and gymnosperms (Peña et al., 2007). Based on side chains branching, xylans can be divided into glucuronoxylan (GX), arabinoxylan (AX), and glucuronoarabinoxylan (GAX; Figure 1). The backbone of xyloglucan is composed of a β-D-(1-4)-glucan with α-D-xylosyl group attached to the 6-position hydroxyl group of approximately 75% of the glucose residues in the skeleton, and some of the 2-position hydroxyl groups of α-D-xylosyl group are additionally connected to β-D-galactosyl or α-l-amylosyl (Figure 1; Perrin et al., 2003). The hemicellulose acetylation, which was covered in our recent review paper (Qaseem et al., 2020), will not be discussed in this article since it does not belong to the sugar substrates. The mannans backbone is inconsistent and can be divided into two categories, one consisting entirely of β-D-mannose and the other also with β-D-glucose, and the side chains of mannans are mainly α-D-galactose linked by α-(1,6) glycosidic chains (Figure 1). Therefore, depending on main chain and side chain glycosyl groups, mannans can be divided into four categories: mannan, galactomannan, glucomannan, and galactoglucomannan (Scheller and Ulvskov, 2010). Β-(1→3, 1→4)-glucans are homogeneous unbranched chain polysaccharides and composed of three or four β-D-glucose units connected by β-1, 3 bonds and β-1, 4 bonds, and the content of triose units is generally higher than that of tetraose units, while the proportion of β-1, 3 and β-1, 4 bonds varies among different sources of β-glucans (Figure 1; Hu et al., 2015; Zielke et al., 2018; Chang et al., 2021).

In addition to the maintenance of cell wall organization, hemicelluloses are important group of cell wall polysaccharides which perform many functions, such as structure of primary and secondary walls, cell expansion, seed storage carbohydrates, and aggregation to facilitate plant growth. All xylan-deficient mutants exhibit collapsed xylem vessels and have severely impaired growth and fertility with decreased mechanical strength in stem, indicating the importance of xylans in secondary wall strengthening (Scheller and Ulvskov, 2010). Some xyloglucans not only have a protective role serves as a physical barrier to prevent pathogens from invading and colonizing, and they protect plant from aluminum toxicity, but also has a vital role in cell wall extension and providing strength to plant organs as it binds along the length of cellulose microfibrils (Park and Cosgrove, 2015; Claverie et al., 2018; Galloway et al., 2018; Wan et al., 2018; Kuki et al., 2020). Recent research has revealed that *Xanthomonas*, the main causal pathogens of citrus bacterial canker disease, has a complicated enzymatic machinery capable of depolymerizing xyloglucans and disrupting the cell wall (Vieira et al., 2021). Function of mannan depends on tissue in which they are present; in cell wall, these have structural role and provide strength and hardness, while in seeds, they function as storage polysaccharides. The specific function of β-(1→3, 1→4)-glucan in plants is not yet clear.

## Nucleotide Sugars Biosynthesis

**Figure 2:**
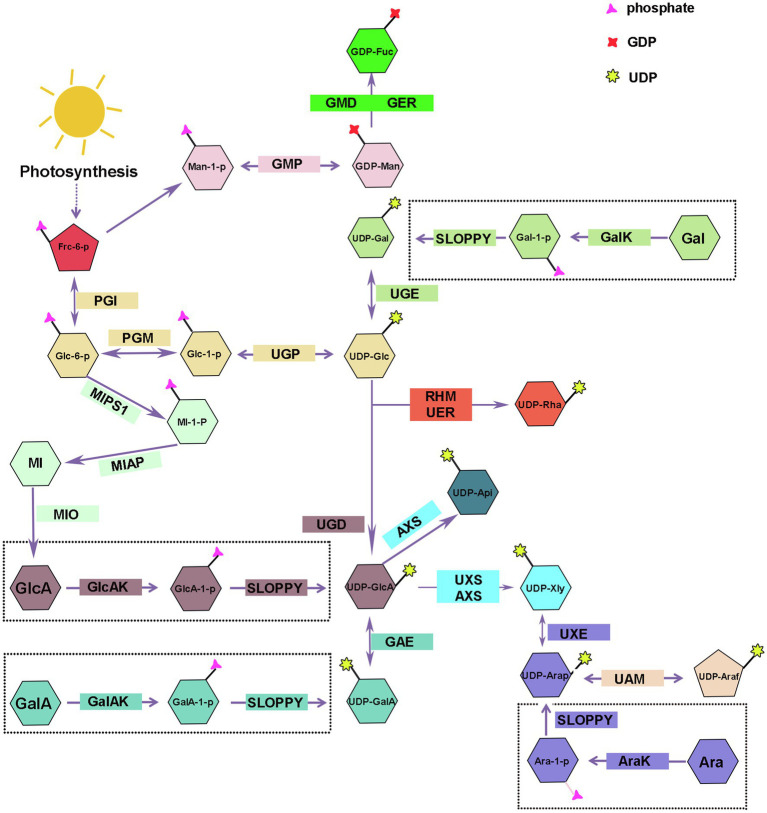
Biosynthesis of nucleotide sugars for plant hemicellulose. Frc-6-P, Fructose-6-phosphate; Glc-1-P, Glucose-1-Phosphate; Man-1-P, Mannose-1-Phosphate; UDP-Glc, uridine diphosphate glucose; UDP-GlcA, UDP-glucuronate; UDP-Api, UDP-Apiose; Xyl, UDP-xylose; UDP-Gal, UDP-galactose; UDP-GalA, UDP-galacturonate; UDP-UDP-Ara, UDP-arabinose; UDP-Rha, UDP-rhamnose; GDP-Man, guanosine diphosphate-mannose; GDP-Fuc, GDP-fucose; UGP, uridine diphosphate glucose pyrophosphorylase; GMP, guanosine diphosphate-mannose pyrophosphorylases; UGE, UDP-galactose/glucose 4-epimerase; GalK, galactokinase; UGD, UDP-D-glucose dehydrogenase; GlcAK, glucuronide kinase; GAE, UDP-GlcA 4-epimerase; GalAK, GalA kinase; UXS, UDP-GlcA decarboxylase; AXS, UDP-D-apiose/UDP-D-xylose synthase; UXE, UDP-Xyl epimerase; UAM, UDP-arabinopyranose mutase; RHM, rhamnose synthase; UER, UDP-4-keto- 6-deoxy-D-glucose 3, 5-epimerase 4-reductase; GMD, GDP-mannose-4, 6-dehydratase; GER, GDP-4-keto-6-deoxymannose-3, 5-epimerase −4-reductase (GDP-fucose synthetase); MIPS1, myo-inositol-1-phosphatase synthase; MI-1-P, myo-inositol-1-phosphatase; MIAP, myo-inositol alkaline phosphatase; MI, myo-inositol; MIO, myo-inositol oxidase.

UDP-nucleotide sugars are glycosyl donors for hemicellulose biosynthesis, and their synthesis is divided into a “*de novo* pathway” and a “salvage pathway” (Figure 2). The majority of nucleotides are synthesized *via* the *de novo* pathway, which involves a series of sugar interconverting enzymes in the cytosol and Golgi (Bar-Peled and O’Neill, 2011). UTP is used by sugar-specific kinases and pyrophosphorylases to specifically convert free monosaccharides, breakdown produced releasing from polysaccharide, into their corresponding UDP-sugars *via* the “salvage” pathway (Kotake et al., 2007; Bar-Peled and O’Neill, 2011). During plant growth and development, some of hemicelluloses are metabolized or remodeled releasing free monosaccharides that gradually accumulated in the plant and finally might result in sugar toxicity (Althammer et al., 2020). SLOPPY, a recombinant protein encoded by Arabidopsis gene *At5g52560*, has a very strong ability to convert GlcA-1-P, Glc-1-P, Gal-1-P, Xyl-1-P, Ara-1-P, and GalA-1-P into their corresponding UDP-sugars (Yang et al., 2009; Decker and Kleczkowski, 2018). At present, we still do not know how many nucleotide sugars are provided by the “salvage” pathway in plants or if the substrate monosaccharides are produced from polysaccharide degradation in the cell wall, cytoplasm, or both (Dhonukshe et al., 2006).

### UDP-Glc and GDP-Man

UDP-Glc and GDP-Man are the starting compounds for the synthesis of all hemicellulose riboside sugars. Fructose-6-phosphate (Fru-6-P), the photosynthetic intermediate, is converted to glucose-1-phosphate (Glc-1-P) and mannose-1-phosphate (Man-1-P) by the collective effect of phosphate-sugar isomerase and metathesis enzymes, and then, uridine diphosphate glucose pyrophosphorylase/guanosine diphosphate-mannose pyrophosphorylases (UGP/GMP) convert reversibly Glc-1-P and Man-1-P into UDP-Glc and GDP-Man in cytosol (Reiter, 2008; Figure 2). Munch Petersen discovered UGP in yeast cells for the first time in 1953. There are two genes encoding UGP in Arabidopsis (Meng et al., 2009) and rice (Long et al., 2017). Silencing *Atugp1/Atugp2* genes in Arabidopsis reduces the activity of UGP and reduces seed yield by 50% (Meng et al., 2009). As the most abundant nucleotide sugars in plants, UDP-Glc can be obtained through two other sources addition to above-mentioned pathways. Sucrose synthase can reversibly catalyze the degradation of sucrose to UDP-glucose and fructose; however, in the presence of UDP, glucose inhibits the reaction in both directions (Ruan et al., 2003; Reiter, 2008; Abdullah et al., 2018). Gal, Glc, and Man produced by the degradation of plant cell wall polysaccharides can also be used as substrates for UDP-Glc (Figure 2). Some plants glycosyltransferases can catalyze the interconversion of sugar molecules between the oligosaccharyl in glucoside and other UDP-monosaccharides to compose UDP-Glc in the presence of UDP (Bode and Muller, 2007; Bar-Peled and O’Neill, 2011).

### UDP-Gal

Galactose is important for the plant growth and occupies a large proportion in a variety of hemicellulose polysaccharides, such as xyloglucan and galactomannan (Scheller and Ulvskov, 2010). There are two mechanisms for the synthesis of UDP-Gal: the *de novo* pathway and the “salvage” pathway.

#### *De novo* Pathway

UDP-galactose/glucose 4-epimerase (UGE) catalyzes the interconversion of UDP-Glc and UDP-Gal, and the reaction is reversible (Figure 2). There are two types of UGE in vascular plants. In addition to catalyzing the conversion of UDP-glucose and UDP-galactose, one type of UGE can also reversibly convert UDP-xylose and UDP-arabinose, and different types of UGE have different catalytic efficiency of UDP-xylose in different plants (Guevara et al., 2014; Yin et al., 2016a). There are five *AtUGE* genes in the Arabidopsis genome, all of them have catalytic activity. AtUGE1 and AtUGE3 mainly catalyze the conversion of UDP-galactose to UDP-glucose, while AtUGE2, AtUGE4, and AtUGE5 mainly catalyze the conversion of UDP-glucose to UDP-galactose (Seifert, 2004). Reverse genetic studies of these five genes revealed no significant phenotypic changes in the single mutant. However, a significant decrease in the galactose content was seen in cell wall of double mutant, indicating that the UGE proteins of different isoforms have functional redundancy and synergy (Seifert, 2004; Rösti et al., 2007) and participate in many physiological processes, such as cell growth and differentiation, cell-to-cell communication, and defense responses by regulating the interconversion of nucleotide sugars (Hou et al., 2021). In addition to Arabidopsis, similar phenomena have been observed in other plants, and including *Oryza sativa* (Kim et al., 2009; Zhang et al., 2020), barley (Zhang et al., 2006), *Phyllostachys edulis* (Sun et al., 2016), and *Ornithogalum caudatum* (Yin et al., 2016a) were reported to contain various plant *UGE* genes. Compared with the wild type, these studies found that the content of galactose and glucose increased in the hemicellulose polysaccharide profile of rice OsUGE1-OX overexpression plants (Guevara et al., 2014). In contrast, *OsUGE2* mutation significantly reduced accumulation of arabinogalactan proteins in the cell walls, which consequently affected plant growth and cell wall deposition (Zhang et al., 2020).

#### “Salvage” Pathway

Using real-time NMR spectroscopy to monitor the enzymatic reaction, the investigators confirmed that Arabidopsis galactokinase (GalK) phosphorylates galactose to Gal-1-P at position C-1. Finally, Gal-1-P is converted to UDP-Gal in the presence of SLOPPY (Yang et al., 2009; Decker and Kleczkowski, 2017; Figure 2). The AtGALK T-DNA insertion mutant (*atgalk*) showed no growth or morphological defects in the absence of Arabidopsis galactokinase and was unable to use free Gal and accumulated it in vegetative tissues; the phenotype was recovered by constitutively overexpressing the *AtGALK* cDNA (Egert et al., 2012). The toxicity of free galactose has yet to be determined, but galactose-1-phosphate or an imbalance in the sugar-1-phosphate and nucleotide sugar network can cause growth defects.

### UDP-GlcA

Glucuronic acid mainly exists in glucuronoxylan and glucuronoarabinolxylan (Figure 1). UDP-GlcA is a key intermediate product in the process of nucleotide sugar metabolism and direct precursor of UDP-GalA, UDP-Api, and UDP-Xyl, and critical substrate for the transformation of UDP-monosaccharides from six-carbon sugars to five-carbon sugars (Bar-Peled and O’Neill, 2011).

#### *De novo* Pathway

UDP-D-glucose dehydrogenase (UGD) action results in the irreversible elimination of hydrogen at the C-6 position, resulting in the conversion of UDP-Glc to UDP-GlcA (Figure 2). Since it was cloned in soybean in 1996 (Tenhaken and Thulke, 1996), the gene encoding UGD has been cloned in various other plants, such as Arabidopsis (Klinghammer and Tenhaken, 2007), cotton (Pang et al., 2010), *Larix gmelinii* (Li et al., 2014), and *moso bamboo* (Yang et al., 2020). Four *UGD* genes are identified in Arabidopsis, which differ in their enzyme kinetic properties and tissue expression specificity, including *AtUGD3* having the highest activity (Klinghammer and Tenhaken, 2007). Yang and his colleagues discovered nine *UGD* genes with three predicted conserved domains, one of which, *PeUGDH4*, was found in the cytoplasm and showed strong expression in the leaf and stem. The overexpression of *PeUGDH4* in Arabidopsis dramatically boosted hemicellulose production and accumulation (Yang et al., 2020). On the other hand, UDP-GlcA can also be formed *via* myo-inositol oxygenase pathway. D-glucose-6-phosphate is cyclized under the action of myo-inositol-1-phosphate synthase to form myo-inositol-1-phosphate, myo-inositol alkaline phosphatase catalyzes the dephosphorylation of later to form myo-inositol, and myo-inositol is oxidized to UDP-glucuronic acid by the action of myo-inositol oxidase (Lorence et al., 2004; Endres and Tenhaken, 2009; Pieslinger et al., 2010; Alford et al., 2012; Figure 2).

#### “Salvage” Pathway

There are two genes in Arabidopsis (*At3g01640* and *At5g14470*) that encode a α-D-glucuronide acid-1-phosphate kinase (GlcAK), which phosphorylates GlcA to GlcA-1-P using ATP and is then pyrophosphorylated by SLOPPY to UDP-GlcA (Geserick and Tenhaken, 2013; Figure 2).

### UDP-GalA

GalA is present in xyloglucan side chain of lower plants, such as mosses, and also part of the tetrasaccharide reducing terminus of dicotyledonous and gymnosperm xyloglucan (Peña et al., 2007; Pena et al., 2008). The research on UDP-GalA synthesis-related proteins in plants started late.

#### *De novo* Pathway

UDP-GalA can reversibly transform from UDP-GlcA *via* UDP-GlcA 4-epimerase (UGLcAE, GAE), and the activity of GAE is NAD^+^-dependent (Figure 2). In 2004, three research teams identified GAE enzymes almost simultaneously and performed expression level analysis. The results showed that there were six *GAE* genes in the Arabidopsis genome, and all of them were localized in the Golgi apparatus and differently expressed in Arabidopsis roots, leaves, pollen, and angiosperms (Gu and Bar-Peled, 2004; Molhoj et al., 2004; Usadel et al., 2004; Rösti et al., 2007). In addition to Arabidopsis, genes encoding GAE were also identified in tomato (Ding et al., 2018), *Nicotiana benthamiana* (Ahmed et al., 2020), and *O. caudatum* (Yin et al., 2016b). UAE isoforms in different plant species have different enzymatic properties, but UAE isoform of the same plant are highly conserved. The GAE can produce UDP-GlcA and UDP-GalA in Arabidopsis, maize, and rice with a ratio of 1:2, and this reversible reaction was inhibited by UDP-Ara and UDP-Xyl, though the degree of GAE inhibition by UDP-Xyl varied among plants (Gu et al., 2009; Figure 2).

#### “Salvage” Pathway

The α-D-galacturonic acid-1-phosphate kinase (GalAK) phosphorylates GalA to GalA-1-P, then GlcA-1-P can be pyrophosphorylated by SLOPPY to UDP-GalA (Decker and Kleczkowski, 2017). Arabidopsis has a single copy of the *GalAK* gene (*At3g10700*), and its catalytic activity was confirmed using real-time NMR (Yang et al., 2009). However, the extent to which UDP-GalA formed by this pathway contributes to the UDP-GalA in plants needs to be further investigated (Bar-Peled and O’Neill, 2011; Figure 2).

### UDP-Xyl

Xylose is a significant component of xyloglucan and xylan. With the participation of NAD^+^ and NADH, UDP-GlcA decarboxylase (UDP-GlcA-DC/UXS) catalyzes the decarboxylation of UDP-GlcA to form UDP-Xyl in an essentially irreversible reaction (Harper and Bar-Peled, 2002; Figure 2). The proteins encoded by the Arabidopsis *UXS* gene family are classified as membrane-anchored or cytoplasmic soluble. *UXS1*, *UXS2*, and *UXS4* are membrane-anchored proteins that are found in the Golgi, whereas *UXS3*, *UXS5*, and *UXS6* are soluble proteins that are found in the cytoplasm (Harper and Bar-Peled, 2002; Kuang et al., 2016). In Arabidopsis xylan synthesis, UXS localized in the cytoplasm plays a more essential role; however, xylosyltransferases all use UDP-Xyl in the Golgi for hemicellulose synthesis, so UDP-Xyl synthesized in the cytoplasm must be transferred to the Golgi (Kuang et al., 2016; Zhao et al., 2018). Ebert et al. (2015) discovered types of UXTs that were localized to the Golgi, and Arabidopsis has three *UXT* genes. Except for the *Atuxt1* mutant, whose xylose concentration was significantly lower than that of the wild type, no other *UXT* mutant showed a clear phenotype (Harper and Bar-Peled, 2002; Ebert et al., 2015). Further, Zhao et al. (2018) identified that *uxt1uxt2uxt3* triple mutants showed an uneven xylem and xylan deposition defects (Zhao et al., 2018). Like Arabidopsis, rice (*O. sativa*) also has six *UXS* genes, which were classified into three types (Suzuki et al., 2003, 2004). Subsequently, besides Arabidopsis and rice, *UXS* gene family with varied members has been cloned from only a few plants, for example, tobacco (*Nicotiana tabacum*; Bindschedler et al., 2007), *Gossypium hirsutum* (Pan et al., 2010), *Populus tomentosa* (Du et al., 2013), and *O. caudatum* (Yin et al., 2016b).

UDP-D-apiose/UDP-D-xylose synthase (AXS) can also produce UDP-Xyl with UDP-GlcA as substrate in plants (Figure 2). Besides, AXS can also convert UDP-GlcA to UDP-Apiose *via* decarboxylation and rearrangement of the carbon skeleton (Figure 2). Arabidopsis has two *AtAXS* genes that are ubiquitously expressed across all tissues and developmental stages, with AtAXS2 showing higher overall expression. AXS has lower enzyme activity to convert UDP-GlcA to UDP-Xyl than UXS, implying that it functions on pectin RG-II side chain A and B biosynthesis by Apiose (Zhao et al., 2020a). Although AXS and UXS can utilize the same substrate, AXS may not have evolved from UXS and both may have their own synthetic precursors (Gu et al., 2010). Under normal physiological conditions in plants, AXS plays a minor role in the synthesis of UDP-Xyl, because its optimum activity conditions close to the pH and temperature in plants (pH 5–6, temperature 20–30°C), while AXS makes a difference in harsh conditions with higher temperature (50°C) and pH optimum (pH 8.5; Yin et al., 2016b).

### UDP-Ara

There are two forms of arabinose, furanose and pyranose. Arabinose is found mostly as furanose in grass xylan, and xyloglucans of pteridophytes and solanaceous plants, and is a major component of the side chains of glucuronide arabinoxylan and arabinoxylan. Pyranose, on the other hand, is thermodynamically more stable and has been detected earlier from various plants as it has been studied more extensively. The synthesis of UDP-Ara*p* also has two ways.

#### *De novo* Pathway

UDP-Xyl epimerase (UXE) can catalyze the allosteric transition of UDP-Xyl into UDP-Ara*p* in a reversible manner (Figure 2). The Arabidopsis genome contains four *UXE* genes, which encode the membrane-bound protein UXE, which is present in the Golgi apparatus. The cell wall arabinose level was drastically reduced in the *AtUXE1* gene mutant *mur4* screened by EMS induction; however, the ability to synthesize UDP-Ara*p* was not completely absent (Burget and Reiter, 1999; Burget et al., 2003). Among the three *UXE* genes in rice, the total expression of *OsUXE1* was significantly higher than *OsUXE2* and *OsUXE3* in mature rice, especially in the middle of the stalk, indicating that *UXE1* plays a critical role in UDP-Ara*p* production in mature rice, and the arabinose content was reduced by 2.19% in the cell wall of the rice *ux1uxe2* double mutant compared with the wild type (Chen et al., 2021).

#### “Salvage” Pathway

Arabinose kinase (AraK) can use Ara as a substrate and phosphorylate it to Ara-1-P, which later can be pyrophosphorylated to UDP-Ara*p* by SLOPPY (Neufeld et al., 1960; Figure 2). There are two *AraKs* in the Arabidopsis genome (*AT4G16130*, *ARA1*; *AT3G42850*, *ARA2*), and *ara1* mutants have lost AraK activity and have diminished metabolism of arabinose (Gy et al., 1998). The free arabinose content in plants increased significantly after mutation of the *ARA1* gene, whereas the *ara2* mutant did not accumulate free arabinose, probably because of the relatively low expression of the *ARA2* gene (Behmüller et al., 2016).

After UDP-Ara*p* is synthesized, UDP-arabinopyranose mutase (UAM) isomerizes it into UDP-Ara*f* in the cytoplasm, which is then transferred to the Golgi apparatus to participate in polysaccharide synthesis (Figure 2). The interconversion of UDP-Ara*p* and UDP-Ara*f* is reversible, and the reaction tends to produce pyranose products. Rice, like Arabidopsis and pea, has three UAM proteins (UAM1-3), with 80% of them localized in the cytoplasm (Klinghammer and Tenhaken, 2007). Willis et al. (2016) used RNAi to downregulate the expression of the *PvUAM1* gene in switchgrass, and the arabinose associated with the cell wall was reduced by more than 50% in the mutant leaves and stems, resulting in a compensatory response with increased cellulose and lignin content (Willis et al., 2016). Honta et al. (2018) used RNAi to downregulate four *NcUAM* genes and found that, compared to the WT, arabinose content was diminished by 35% in NtUAM-KD cell walls (Honta et al., 2018).

### UDP-Rha

Rhamnose, also known as 6-deoxy-l-mannose, is used in the synthesis of the reduced tetrasaccharide terminus of xylan in dicotyledonous and gymnosperm (Peña et al., 2007; Jiang et al., 2021). The conversion of UDP-Glc to UDP-Rha in bacteria requires three enzymatic sequences: dehydratase, isomerase, and reductase, while in plants, it require rhamnose synthase (RHM) and UDP-4-keto-6-deoxy-D-glucose 3, 5-epimerase 4-reductase (NRS/UER; Watt et al., 2004; Oka et al., 2007; Figure 2). RHM possesses three enzymatically active structural domains (RHM1; RHM2; RHM3) and catalyzes the three-step reaction of the substrate UDP-glucose to produce UDP-Rha in the presence of cofactors NAD^+^ and NADPH (Reiter and Vanzin, 2001; Oka et al., 2007). *AtRHM1* was found practically everywhere, with higher levels of expression in roots and cotyledons. Overexpression of the *AtRHM1* gene in Arabidopsis resulted in a 40% increase in rhamnose content in the cell wall as compared to the wild type (Wang et al., 2009). One or more *RHM* genes were found in a variety of plants, including *Lobelia erinus* (Hsu et al., 2017), *Camellia sinensis* (Dai et al., 2018), prunes, and peaches (Zhao et al., 2020b), and the recombinant proteins were shown to catalyze the production of UDP-rhamnose from UDP-glucose by enzymatic analysis and *in vitro* enzymatic reaction.

The At1g6300 gene, which encodes UDP-4-keto-6-deoxy-D-glucose3,5-epimerase/UDP-4-keto-rhamnose 4-keto-reductase (NRS/UER), was also discovered in Arabidopsis, and the amino acids encoded by AtNRS/UER are highly similar to the C-terminal sequence of AtRHM2 amino acids, with both isomerase and reductase (Watt et al., 2004). The fusion enzyme (VvRHM-NRS) may convert UDP-glucose to UDP-rhamnose by fusing the N-terminus of VvRHM with the bifunctional (NRS/UER) from Arabidopsis. However, it is unclear how NRS/UER function *in vivo* (Pei et al., 2018).

### GDP-Fuc

Fucose is mainly present in pectin and seed coat mucilage, less in hemicellulose, and only the side chain of xyloglucan contains a small amount of GDP-Fuc (Pena et al., 2008; Bar-Peled and O’Neill, 2011).

GDP-Fuc is converted from GDP-man, which involves three sequential enzyme processes, just like the conversion from UDP-Glc to UDP-Rha. GDP-mannose-4, 6-dehydratase (GMD) is a key enzyme in the GDP-fucose synthesis pathway, catalyzing the formation of GDP-4-keto-6-deoxy-D-mannose from GDP-D-mannose, and then, in the presence of NADPH, the GDP-4-keto-6-deoxymannose-3, 5-epimerase-4-reductase (GDP-fucose synthetase, GER) catalyzed the conversion of the intermediate into GDP-Fuc (Figure 2). GER contains two enzymatic activity domains, epimerase and reductase, and it has been shown that this system can catalyze epimerism of substrates even in the absence of NADPH, indicating that epimerism and reduction reactions are carried out independently (Menon et al., 1999). At1g73250 (GER1) and At1g17890 (GER2) encode GER isoforms with 88 percent sequence similarity (Bar-Peled and O’Neill, 2011). In Arabidopsis, two genes, that is, *GMD1* and *GMD2* (*MUR1*), encode for *GMD*, with *GMD2* being the major housekeeping gene and expressed in most cell types of the root, while *GMD1* is expressed in the root tip, juvenile stipule organs, and pollen grains (Bonin et al., 2003). Arabidopsis *mur1* mutant lacks GMD2 in the aboveground portion and has almost no fucose in the cell wall, and biochemical assays indicate that the nucleotide sugar conversion is blocked in the first step, and the GMD is mutated (Bonin et al., 1997; Bonin and Reiter, 2000; Freshour et al., 2003). Compared to wild-type plants, 80 percent of *N. benthamiana* plants with GMD repression using virus-induced gene silencing (VIGS) and RNA interference (RNAi) were fucose-free in total soluble protein (Matsuo and Matsumura, 2011).

## Transport of Nucleotide Sugars For Plant Hemicellulose

**Figure 3:**
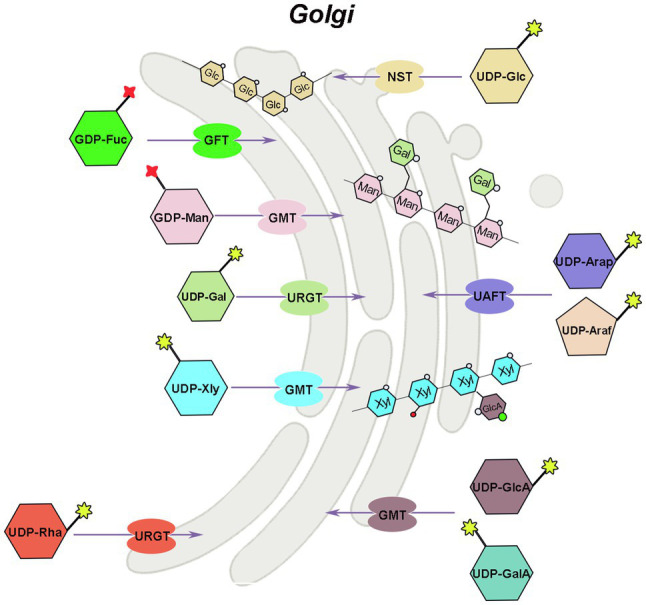
Mechanism of hemicellulose substrate transport. UTR, UDP-galactose/UDP-glucose transporter; URGT, UDP-Rha/UDP-Gal transporter; UAFT, UDP-arabinofuranose transporter; UXT, UDP-Xyl transporter; UAfT, UDP-Araf transporter proteins; GFT, GDP-fucose transporter; GMT, GDP-mannose transporter.

Hemicellulose is synthesized by glycosyltransferases in the Golgi apparatus, which are type II transmembrane proteins with functional structural domains in the Golgi lumen (Reyes and Orellana, 2008). The majority of nucleotide sugar synthases and all salvage pathway-related enzymes are found in the cytoplasm, whereas GAE, UXE, and a portion of UXS are found in the Golgi apparatus, that is, place where hemicellulose synthesis occurs. The related glycosyltransferases can directly use the nucleotide sugars produced in the Golgi apparatus (GTs). However, the nucleotide sugars in the cytoplasm must therefore be transported to the Golgi apparatus to added to specific polysaccharide acceptors and participate in hemicellulose synthesis (Figure 3). Because the phosphate groups in nucleotide sugars have a high molecular mass (500–650Da) and a negative charge, they cannot diffuse directly across membranes and must be transported by nucleotide sugar transporters (NST), which are antiporters that exchange nucleoside monophosphate for specific NDP-sugars (Orellana et al., 2016; Figure 3). NST generally contains 300–350 amino acids and has 6–10 transmembrane domains (Handford et al., 2006). Plant NSTs belongs to the nucleotide sugar transporter/triose phosphate translocator (NST-TPT) super-family, and the *NST-TPT* gene family of Arabidopsis has 51 members, which can be divided into six clades and are generally highly substrate-specific (Knappe et al., 2003; Rautengarten et al., 2014). Except for UDP-GalA and UDP-Arap, all other transport proteins of hemicellulose substrate nucleotide sugars have been identified in a variety of plants, including Arabidopsis (Baldwin et al., 2001; Norambuena et al., 2002, 2005; Knappe et al., 2003; Handford et al., 2004, 2012; Rollwitz et al., 2006; Rautengarten et al., 2008, 2011, 2014; Mortimer et al., 2013; Saez-Aguayo et al., 2017), rice (Zhang et al., 2011), tobacco, grapevine (*Vitis vinifera* L.), and *Dendrobium officinale* (Yu et al., 2018).

There are two ways to identify the function of NST, the most direct way is to analyze its biochemical activity, and the other way is to screen for mutants of the *NST* gene. Joshua Heazlewood’s team at the University of Melbourne has developed a rapid method for measuring NST biochemical activity, and in combination with Arabidopsis mutant analysis, the function of individual genes was rapidly unraveled in the NST-TPT family (Rautengarten et al., 2014, 2017; Saez-Aguayo et al., 2017). Substrate-specific examination of plant NSTs indicated that certain NSTs can transport two or more UDP-sugars, and many NSTs maintain their ability to transport either UDP-glucose or UDP-galactose (Orellana et al., 2016).

The AtUTr1 gene that transports both UDP-Gal and UDP-Glc was discovered in 2000 by Norambuena et al. (2002; Figure 3). The tobacco plant expressing human UDP-galactose transporter gene 1 (*hUGT1*) showed significantly higher galactose to total monosaccharide ratios in the hemicellulose and pectin fractions of transgenic plants compared to control plants, enhanced growth, and increased chlorophyll and lignin accumulation (Abedi et al., 2016). There are six UDP-Rha/UDP-Gal transporter (URGT) in the Arabidopsis genome, which are localized to the Golgi apparatus, and all have detectable transporter activity, while mutants of *URGT2* gene have significantly reduced Rha content in the seed coat mucilage, and URGT1 and URGT2 overexpressing Arabidopsis have significantly increased Gal content in their cell walls (Rautengarten et al., 2014; Figure 3). The upregulation of UDP-arabinofuranose transporter protein (UAFT2) suggests the existence of compensatory mechanisms triggered by URGT2 deficiency, and URGT2 overexpression in *urgt1* mutant rescues reduced galactose in Arabidopsis rosette leaves (Parra-Rojas et al., 2019; Celiz-Balboa et al., 2020). In addition, it has also been shown that the UDP-Gal transporter, named AtUTr2, is located in the Golgi apparatus and is highly expressed in the root and calli (Norambuena et al., 2005). The nucleotide sugar transporter (GONST1) localized to the Golgi apparatus in Arabidopsis was identified as the GDP-mannose transporter (GMT; Baldwin et al., 2001; Figure 3). Yu et al. (2018) also cloned three *DoGMT* genes in *Dendrobium* which are mainly expressed in the stem (Yu et al., 2018). In Arabidopsis, a UUAT1 gene was identified, which produces a Golgi-localized protein that transports UDP-GlcA and UDP-GalA *in vitro* (Saez-Aguayo et al., 2017). There are three UDP-Xyl transporters (UXT; *UXT1*, AT2G28315; *UXT2*, AT2G30460; and *UXT3*, AT1G06890) in the Arabidopsis genome. Mutants of the *UXT1* gene have significantly reduced Xyl content in the cell wall, and triple mutant exhibits collapsed vessels and reduced cell wall thickness and significantly affected xylan content and fine structure (Ebert et al., 2015; Saez-Aguayo et al., 2017; Zhong et al., 2017). The discovery of the UXT genes and the research results on the *uxt* mutants suggest that the UDP-Xyl in the cytoplasm is very essential for the growth and development of Arabidopsis. Four genes in the Arabidopsis NST family encode UDP-Ara*f* transporter proteins (UA*f*T) localized in the Golgi apparatus (Figure 3). Compared with the wild type, the phenotype of the *uaft* mutant did not change significantly, but the Ara content in the cell wall of the *uaft4* mutant leaves was decreased (Rautengarten et al., 2017). GDP-fucose transporter (GFT), which can import GDP-fucose into the Golgi, has now been identified from *Phaeodactylum tricornutum* and Arabidopsis (Rautengarten et al., 2016; Zhang et al., 2019). The GFT1-silenced plants exhibited severe growth inhibition or even death, with up to 80% decrease in fucose content in cell wall-derived xyloglucan and rhamnogalacturonan II (Rautengarten et al., 2016).

## Nucleotide Sugars in Hemicellulose Biosynthesis

Forward and reverse genetic approaches, as well as biochemical enzyme analyses, have recently made significant advances in hemicellulose biosynthesis. Pair genes of *IRX9/IRX9L*, *IRX14/IRX14L*, and *IRX10/IRX10L* involved in xylan backbone elongation by added substrate UDP-Xyl in the Golgi (Brown et al., 2007, 2009; Wu et al., 2009, 2010; Hornblad et al., 2013; Jensen et al., 2014). *FRA8*, *PARVUS*, and *IRX8* mainly participate in reducing end biosynthesis by adding substrate UDP-Xyl, UDP-Rha, and UDP-GalA to the xylan backbone (Brown et al., 2007, 2009; Wu et al., 2009). Likewise, five glucuronic acid substitution of xylan (*GUX*) genes are involved in side chain decoration and catalyze the attachment of UDP-GlcA and other nucleotide sugars to the xylan backbone (Lee et al., 2012; Rennie et al., 2012; Bromley et al., 2013).

The β-1,4-glucan synthase, α-1,6-xylosyltransferase, β-1,2-galactosyltransferase, and α-1,2-fucosyltransferase play primary role in xyloglucan biosynthesis, and the former synthesizes the glucan backbone and different types of glycosyl transferases produce the broad diversity of XyG side chain to decorate the glucan chain (Zabotina, 2012). *CSLC4* gene from *GT2* family encodes for β-1,4-glucan synthase, which enzyme synthesis of xyloglucan backbone with UDP-Glc as substrate, and α-1,6-xylosyltransferase encoded by five genes of *GT34* family, *XXT1-5*, also involved in xyloglucan backbone synthesis by affixing UDP-Xyl (Faik et al., 2002; Cocuron et al., 2007; Liepman and Cavalier, 2012; Vuttipongchaikij et al., 2012). *MUR3*, *XLT2*, *XUT1*, and the *XSTs* are part of the same subclade of *GT47* involved in xyloglucan synthesis or side chain decoration by substituting two different UDP-Xyl residues for UDP-Glc or other nucleotide sugars (Zabotina, 2012; Jensen et al., 2014).

The *CSLD* gene family and *GT2* family members, *CSLA2*, *CSLA7*, and *CSLA9*, are involved in mannan biosynthesis (Dhugga et al., 2004; Liepman et al., 2005; Verhertbruggen et al., 2011). The recombinant CSLA protein catalyzes the production of mannans when GDP-Man is used as a substrate, and the same protein produces glucomannan with the substrate of a mixture of GDP-Man and GDP-Glc (Liepman et al., 2005). The Csl family of CslF and CslH proteins is the major components of β-(1→3, 1→4)-glucan synthase, and each of them can independently involve in the biosynthesis of the later linking multiple UDP-Glc with β-1→3 or β-1→4-glycosidicor, while CslF proteins and CslH proteins do not need to be active at the same time (Burton et al., 2006; Doblin et al., 2009; Chang et al., 2021).

## Summary and Perspectives

The hemicellulose content and composition vary with different plant species or within the same plant during different growth phases, tissues, and cell types, so do their nucleoside substrates. Many genes involved in the biosynthesis and transport of the substrate nucleotides sugars for hemicellulose were studied *in vitro* and *in vivo*. However, the absorption and utilization of nucleoside sugars are a balance. Multiple nucleoside sugars may be affected if one gene or one substrate is changed or mutated. So, we must employ a systemic approach to investigate nucleotide sugar changes using high-throughput multi-omics analysis, such as transcriptome-proteome-metabolism analysis. It is also unknown which transcription factors regulate nucleotide sugar synthesis networks and how they do so. These researches will provide a better insight into the interconversion and regulation of hemicellulose substrates. As research progressed, researchers found that NST is substrate-specific and can only transport one or two nucleotide sugars specifically. With the continuous development of live cell imaging technology, the spatial and temporal resolution of *in vivo* observation has been greatly improved, making it possible to track the transport process of NST in real time, which can provide a clearer understanding of how NSTs, such as GMT and UAFT, transport nucleotide sugars.

The regarding interconversion of nucleotides sugars and hemicellulose synthesis needs to be further explored in depth. At the same time, CRISPR/CAS9 gene editing technology can be used to knock out nucleotide sugars biosynthesis and transport genes in plants, besides Arabidopsis, to alter the composition and structure of hemicellulose and improve hemicellulose and biofuel utilization in future.

## Author Contributions

WZ drafted the manuscript. WQ constructed figures. HL searched the literature and provided suggestions for writing. AW conceived the project and gave suggestions on the revision of the manuscript. All authors contributed to the article and approved the submitted version.

## Funding

This work was supported by China and the National Natural Science Foundation of China (Grant Numbers 31870653, 31670670, and 31811530009).

## Conflict of Interest

The authors declare that the research was conducted in the absence of any commercial or financial relationships that could be construed as a potential conflict of interest.

## Publisher’s Note

All claims expressed in this article are solely those of the authors and do not necessarily represent those of their affiliated organizations, or those of the publisher, the editors and the reviewers. Any product that may be evaluated in this article, or claim that may be made by its manufacturer, is not guaranteed or endorsed by the publisher.
